# Recurrent Erysipelas: Clinical Challenges and Strategies for Prevention—A Narrative Literature Review

**DOI:** 10.3390/biomedicines13102448

**Published:** 2025-10-08

**Authors:** Dominika Maria Jaskóła-Polkowska, Krystian Blok, Anna Skibińska, Andrzej Chciałowski

**Affiliations:** Department of Internal Medicine, Infectious Diseases and Allergology, Military Institute of Medicine-National Research Institute, 128 Szaserów Street, 04-141 Warsaw, Poland; kblok@wim.mil.pl (K.B.); askibinska@wim.mil.pl (A.S.); achcialowski@wim.mil.pl (A.C.)

**Keywords:** recurrent erysipelas, risk factors, lymphedema, antibiotic prophylaxis, prevention strategies

## Abstract

Recurrent erysipelas is a common and clinically significant condition that poses challenges for both patients and healthcare systems. Each episode may damage lymphatic vessels, leading to chronic lymphedema, which perpetuates the risk of further relapses. Recurrence rates remain high, ranging from 11% in outpatients during the first year to up to 46% of hospitalized patients within three years. The lower limbs are the most frequent site, although recurrences may also occur in other regions, such as the upper limb after mastectomy with lymph node dissection. This review summarizes current knowledge on risk factors, preventive measures, and chemoprophylaxis in recurrent erysipelas. Modifiable risk factors such as obesity, diabetes, venous insufficiency, tinea pedis, and poor hygiene play an important role, while non-modifiable factors include age, sex, and a history of prior episodes. Non-pharmacological strategies—weight reduction, glycemic control, smoking cessation, compression therapy, and meticulous skin care—form the cornerstone of prevention and may reduce the need for long-term antibiotics. Antibiotic prophylaxis, most commonly with oral penicillin V or intramuscular benzathine penicillin, has been shown to lower recurrence rates. However, efficacy may be reduced in patients with chronic edema or severe obesity. Macrolides serve as alternatives in penicillin-allergic patients, but concerns remain about resistance, adverse effects, and drug–drug interactions. In conclusion, recurrent erysipelas requires a multifaceted approach. While antibiotic prophylaxis is effective, its long-term success depends on simultaneous management of underlying conditions. Further studies are needed to define optimal regimens, treatment duration, and non-antibiotic alternatives.

## 1. Introduction

Erysipelas is a bacterial infection of the skin and subcutaneous tissue, involving the upper dermis and lymphatics. The name ‘erysipelas’ originates from Greek, literally translating to ‘red skin’, which accurately reflects the disease’s distinctive red, inflamed appearance. Erysipelas is a notable contributor to hospitalizations due to skin infections in both Europe and the United States.

Erysipelas has a worldwide distribution, occurring sporadically across different populations. The incidence is slightly higher in women than in men. In 2023, Poland reported 5350 cases of erysipelas, translating to an incidence rate of 14.24 cases per 100,000 inhabitants [1]. Erysipelas and cellulitis incidence is increasing, with an estimated 200 cases per 100,000 people per year in European countries [2]. A rising trend in hospitalizations for cellulitis/erysipelas has been observed globally and in Europe, likely driven by demographic shifts towards an aging population and increasing rates of obesity and chronic conditions, including diabetes, venous insufficiency, and lymphedema [3,4]. Although incidence data often combine cellulitis and erysipelas, the two conditions differ clinically; in this review, we focus on erysipelas, while acknowledging that many studies report them together.

Limb erysipelas is typically caused by *Streptococcus pyogenes*(Group A) and other beta-hemolytic streptococci, including Groups B, C, and G, which may be equally prevalent. *Staphylococcus aureus* is a less common cause, though it is more typically associated with purulent skin infections. Facial erysipelas is most commonly attributed to *Streptococcus pyogenes* (Group A), though other streptococcal groups and *Staphylococcus aureus*, including methicillin-resistant *Staphylococcus aureus* (MRSA), have also been reported. The rising incidence of antibiotic-resistant bacteria, such as MRSA, further complicates treatment, underscoring the need for judicious antibiotic use and alternative therapeutic strategies. The onset of erysipelas often follows a disruption in skin integrity, even a minor wound, which facilitates direct invasion of the lymphatic vessels and subsequent spread in the skin.

**Figure 1:**
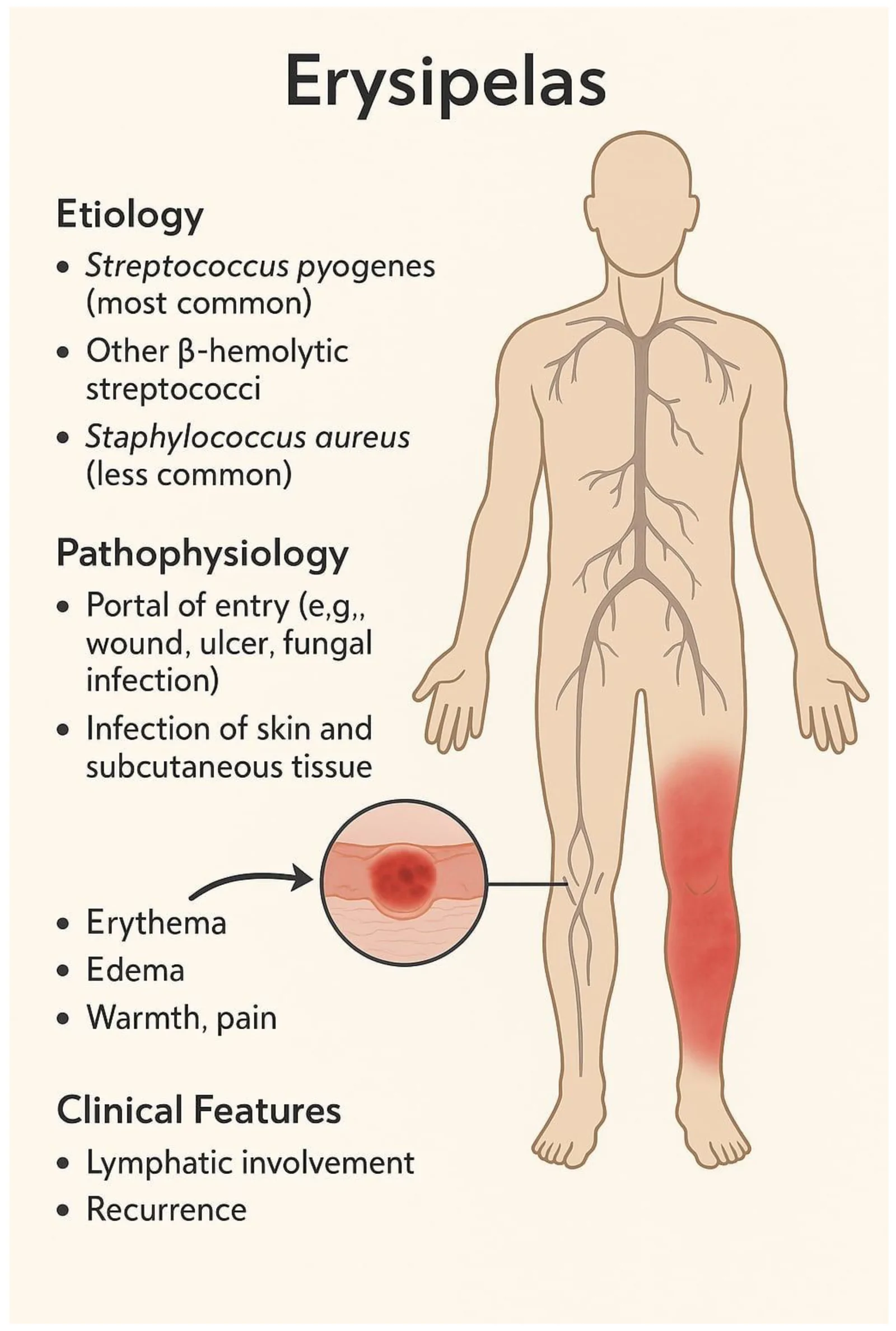
Overview of erysipelas: etiology, pathophysiology, and clinical features. Illustration created with the assistance of ChatGPT (GPT-5, OpenAI).

Portals of entry for erysipelas include insect bites, stasis ulcerations, surgical incisions, and venous insufficiency. Facial cases may also arise from recent nasopharyngeal infections. Predisposing factors include obesity, lymphedema, tinea pedis, leg ulcers, eczema, intravenous drug use, poorly controlled diabetes, and liver disease. Erysipelas of the upper limb in women can be a complication following mastectomy with lymph node dissection. An overviewof erysipelas is presented in Figure 1. Risk factors also contribute to recurrences, with obesity and lymphedema being the most significant factors associated with recurrent episodes. Each recurrence causes further lymphatic damage, creating a vicious cycle that makes subsequent recurrences increasingly likely. Erysipelas often begins with systemic symptoms such as malaise, fever, and chills, which can precede the skin lesion by up to 48 h. Local signs and symptoms include pain, edema, warmth, redness, and a shiny, stretched skin surface. Severe cases may manifest with vesicles, bullae, and tissue necrosis. The condition can be difficult to differentiate from cellulitis, and a conclusive diagnosis may not always be possible. Erysipelas is distinguished from cellulitis by its sharply demarcated borders and faster development, whereas cellulitis tends to have more diffuse borders and a slower onset. Erysipelas is a potentially serious infection, but it is rarely fatal. Penicillin-based antibiotic therapy is the treatment of choice for erysipelas, while macrolides, clindamycin, and fluoroquinolones serve as suitable alternatives in patients with penicillin allergy. Recovery from erysipelas does not confer lasting immunity, and individuals, especially those with predisposing risk factors, may experience repeated infections [5]. This lack of long-term immunity explains why recurrent infections are common, particularly in patients with underlying risk factors.

Erysipelas can result in a range of local complications, including recurrent infections, abscesses, cellulitis, skin necrosis, superficial thrombophlebitis, chronic lymphedema (elephantiasis nostras), scarring, and limb deformity. Systemic complications can also occur, such as sepsis, endocarditis, arthritis, and osteomyelitis. Chronic lymphedema is a particularly significant issue, as damage to lymphatic vessels can lead to persistent fluid accumulation in tissues, increasing the risk of future erysipelas episodes [1]. Chronic lymphedema is not only a complication but also a major risk factor for recurrent erysipelas.

**Figure 2:**
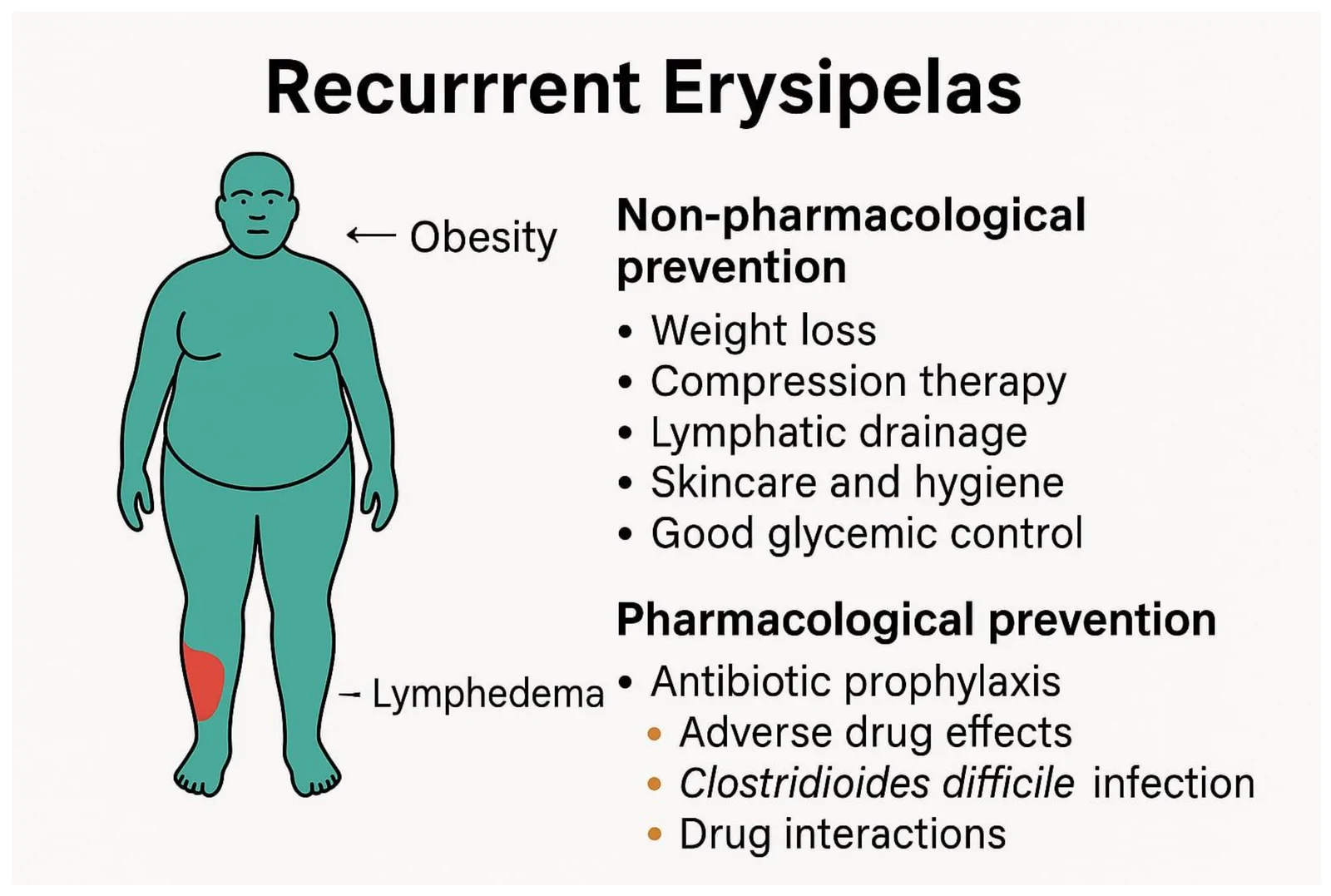
Recurrent erysipelas: major risk factors and preventive strategies. Non-pharmacological prevention includes weight loss, compression therapy, lymphatic drainage, skincare and hygiene, and good glycemic control. Pharmacological prevention relies on antibiotic prophylaxis, which carries potential risks such as adverse drug effects, Clostridioides difficile infection, and drug interactions. Red color symbolize erysipelas. Illustration created with the assistance of ChatGPT (GPT-5, OpenAI).

Given the significant clinical and economic burden associated with recurrent erysipelas, this condition has emerged as a critical area of research and clinical focus. This review aims to provide a comprehensive overview of the current understanding of recurrent erysipelas, including its epidemiology, risk factors, pathophysiology, and existing preventive strategies, while also highlighting their limitations. With the rising prevalence of obesity and other lifestyle-related disorders that increase susceptibility to recurrent erysipelas, the incidence of this condition is expected to grow, underscoring the urgent need for effective preventive and therapeutic strategies. Although a considerable body of literature exists on erysipelas and cellulitis, most studies focus on the first episode and its treatment, while much less attention has been given to the prevention of recurrences. This gap is clinically important, as each new episode of erysipelas causes additional damage to the lymphatic vessels, thereby increasing susceptibility to subsequent recurrences in the same anatomical region. Recurrent disease is particularly common in patients with major risk factors such as obesity and lymphedema; the latter may also occur in cancer survivors after mastectomy with lymph node dissection, a growing clinical population. Despite the high prevalence and burden of recurrent erysipelas, the amount of data specifically addressing this condition remains limited, and there are no uniform international guidelines defining the optimal duration of chemoprophylaxis, the precise timing of its initiation, or the preferred antibiotic choice. Moreover, antibiotic prophylaxis itself is associated with important challenges, including potential adverse effects, drug–drug interactions, disruption of the microbiota, and the risk of Clostridioides difficile infection (CDI) and antimicrobial resistance. These limitations highlight the need to also address underlying risk factors through lifestyle medicine approaches, including the prevention and treatment of obesity, smoking cessation, and optimal management of comorbidities such as diabetes. Major risk factors and preventive strategies of recurrent erysipelas are presented in Figure 2.

Beyond the individual level, recurrent erysipelas carries a significant clinical and socioeconomic burden, leading to pain, impaired quality of life, work absenteeism, hospitalizations, and in severe cases complications such as sepsis or even death. Against this background, the present review aims to synthesize and critically discuss the available evidence on recurrent erysipelas—its epidemiology, risk factors, pathophysiology, and preventive strategies—while underlining existing gaps and highlighting directions for future research and clinical practice. To prepare this narrative review, we performed a literature search in PubMed and Google Scholar using the terms “recurrent erysipelas,” “cellulitis recurrence,” “antibiotic prophylaxis erysipelas,” and “risk factors erysipelas”. Only articles in English were included. In addition, standard textbooks of internal medicine were consulted for background information regarding the first episode of erysipelas. Relevant studies were selected and synthesized to provide a comprehensive and critical overview of the topic. We hypothesize that recurrent erysipelas, though common and clinically significant, remains underrecognized in research and guidelines. By synthesizing the limited available evidence, this review highlights the unmet need for standardized recommendations and integrated preventive strategies.

## 2. Recurrent Erysipelas—Definition and Clinical Context

There is no universally accepted definition of recurrent erysipelas, and the criteria vary considerably across the literature and clinical guidelines. According to the National Institute for Health and Care Excellence (NICE) guideline, recurrent cellulitis is defined as at least two distinct, documented episodes within a 12-month period [6]. The Australian Lymphology Association and the British Lymphology Society (BLS) use a similar threshold of two or more episodes per year, provided that predisposing factors are adequately addressed [7]. The Infectious Diseases Society of America (IDSA) and the South Korean guideline for skin and soft tissue infections (SSTI) apply strict criteria of 3–4 episodes annually despite management of risk factors [7]. In contrast, the Sanford Guide considers two episodes within three years sufficient to indicate frequent recurrence [8]. Clinical studies have also used heterogeneous criteria: Inghammar et al. defined recurrence as more than one episode irrespective of timing [4], while Li et al. required recurrences to occur in the same anatomical location to be considered as such [9]. In a recent Polish retrospective analysis, recurrent erysipelas accounted for 75.7% of cases, although no specific time-based threshold was provided [10].

This wide heterogeneity complicates direct comparison of recurrence rates across studies and highlights the lack of standardization in both research and clinical practice. Some guidelines, such as those cited by Ong, recommend considering antibiotic prophylaxis when patients experience two or more episodes within 12 months, recognizing that such frequency reflects a high-risk population in whom prophylactic strategies may be justified [7].

In clinical practice, recurrent episodes most often occur in the same anatomical location, which is consistent with the underlying lymphatic damage described in the literature. However, patients with bilateral lymphedema or multiple predisposing conditions may develop erysipelas in different sites over time. This raises further uncertainty as to whether a strict requirement that recurrence must occur in the identical anatomical area, as suggested by some authors, is clinically appropriate or universally applicable. Definitions of recurrent erysipelas are summarized in Table 1.

Cellulitis is a local infection of the deep dermis and subcutaneous tissue, most commonly caused by *Staphylococcus aureus* and *Streptococcus pyogenes* [11]. Clinically, cellulitis usually presents as a poorly demarcated lesion with induration, whereas erysipelas is more sharply demarcated and elevated. However, in clinical practice, it is often difficult to distinguish between the two conditions, and in some cases, they may even coexist [12]. It is believed that each episode of erysipelas causes damage to the lymphatic vessels, promoting lymphedema (the abnormal accumulation of lymph in the interstitium), which in turn increases the risk of subsequent recurrences of erysipelas or cellulitis [8,11]. For this reason, in the majority of cases, recurrent episodes occur in the same anatomical location as the initial infection [4]. Some reports indicate that up to 95% of recurrences involve the same site as the preceding episode [13]. Nevertheless, this is not an absolute rule, and episodes may occasionally appear in a different location—for example, on the contralateral lower limb—particularly in patients with bilateral lymphedema or other additional risk factors. Still, most recurrences develop in the same area. The lower extremities are the most frequent site of recurrent erysipelas, largely because lymphedema and other factors that impair local defense against infection commonly affect the legs [4]. In women after mastectomy with lymph node dissection, recurrent erysipelas may instead involve the upper limb due to postoperative lymphedema. Other conditions predisposing to lymphedema include chronic venous insufficiency and morbid obesity [Body Mass Index (BMI) > 50 kg/m^2^], with underlying mechanisms such as increased lymphatic load, dysfunctional lymphatic vasculature, and chronic inflammation [14]. Recurrence rates are substantial. Studies have reported that 25% to as many as 46% of patients hospitalized for erysipelas experience a recurrence within three years. Among those managed in outpatient care, 11% had a recurrence during the first year [4]. Other data suggest that up to 41% of patients may develop recurrence within five years, highlighting the considerable burden of this condition [2]. Therefore, it is essential to identify and address risk factors and to implement effective preventive strategies, both non-pharmacological and pharmacological, to reduce the risk of recurrence and its potential systemic complications.

## 3. Risk Factors of Recurrent Erysipelas

Recurrent erysipelas is associated with various risk factors, including non-modifiable ones such as age and sex, and modifiable ones such as lymphedema, obesity, diabetes, venous insufficiency, cancer, immunodeficiency, skin trauma, and dermatological conditions. Notably, a history of previous erysipelas episodes, especially those causing lymphatic damage, significantly increases the risk of recurrence. Chronic edema constitutes a major risk factor for the development of cellulitis, as it impairs tissue viability by compromising cellular nutrition and oxygenation. Prolonged edema can lead to chronic inflammation, accumulation of cellular debris, and subsequent fibrosis and lymphatic dysfunction, thereby increasing the risk of ulceration and infection. The lymphatic system plays a crucial role in removing excess interstitial fluid and macromolecules; however, in chronic edema, it can become overwhelmed and dysfunctional. Uncontrolled lymphedema can lead to progressive fibroadipose tissue deposition, resulting in a condition that is difficult to treat. The etiology of chronic edema is multifactorial, with primary causes including lymphedema, venous insufficiency, obesity, and immobility. Edema is a prevalent condition, affecting up to 38% of hospitalized patients. Furthermore, it is estimated that over one-third of patients with chronic edema will experience recurrent cellulitis, with the risk escalating in proportion to the severity of edema. Obesity is a multifaceted condition associated with a range of comorbidities and serves as a significant risk factor for cellulitis through various mechanisms. Individuals with obesity are prone to developing skin infections and chronic edema, which can further exacerbate their condition. Obesity adversely affects lymphatic function and lymph node architecture, thereby increasing susceptibility to skin infections. Furthermore, cellulitis in obese patients is often associated with unfavorable outcomes, including an increased risk of treatment failure, underscoring the need for tailored management strategies. Diabetes mellitus is a complex systemic disease characterized by multisystem involvement and increased susceptibility to infections. The condition is often accompanied by comorbidities such as obesity and tinea pedis, which further elevate the risk of recurrent cellulitis. Diabetic complications, including peripheral neuropathy and microvascular disease, can lead to ulcer formation, thereby increasing the likelihood of infection. Moreover, suboptimal glycemic control has been linked to an increased risk of developing cellulitis, highlighting the importance of effective diabetes management in mitigating infectious complications. Chronic venous disease, characterized by venous stasis dermatitis, can present similarly to cellulitis, particularly in cases of bilateral involvement. Sustained venous hypertension and chronic inflammatory changes in dermal tissues, resulting from venous insufficiency, can also lead to lipodermatosclerosis, skin ulceration, and chronic edema. A history of deep vein thrombosis is a significant risk factor for recurrent cellulitis, likely due to the resulting venous insufficiency. Cancer patients are at increased risk of recurrent cellulitis. The primary risk factor is likely edema resulting from tumor invasion, lymph node resection, or radiation therapy. Lymphedema affects over 25% of patients with breast cancer. Additionally, neutropenia occurring in immunodeficiency increases the risk of infections caused by unusual fungal pathogens and more severe invasive bacterial skin infections. Chronic wounds and ulcers provide a conduit for pathogenic bacteria to enter the body. The formation of bacterial biofilms in chronic wounds can impede the healing process and increase the risk of infection. In certain cases, particularly with deeper wounds, tissue samples may be necessary to guide effective treatment of infected ulcers. Chronic dermatomycosis, especially tinea pedis and onychomycosis, increases the risk of recurrent cellulitis. The interdigital space can harbor pathogenic bacteria, including group A streptococcus. Recurrence rates can reach 25%, particularly in patients with diabetes or a family history. Other risk factors include psoriasis and lower leg vein surgery [7]. Risk factors are summarized in Table 2.

## 4. Non-Pharmacological Strategies for the Prevention of Recurrent Erysipelas

Since a single episode of erysipelas or cellulitis is frequently followed by recurrence, it is essential to address risk factors and apply preventive measures. Prevention of recurrent erysipelas can be divided into non-pharmacological strategies and pharmacological (i.e., chemoprophylaxis). Some risk factors for recurrence are modifiable through lifestyle or medical interventions, whereas others are not. Obesity is one of the most importantmodifiable risk factors. While in some cases it may result from genetic predisposition or chronic corticosteroid therapy, in most patients it is linked to sedentary behavior, caloric excess, and insufficient physical activity. Obesity itself is strongly associated with type 2 diabetes mellitus, which contributes to impaired immune function, reduced phagocytic activity, and delayed wound healing, thereby creating additional portals of entry for pathogens [7]. Lifestyle medicine—healthy diet, regular physical activity, and strict glycemic control—is therefore central to recurrence prevention. Emerging obesity medicine (obesitology) provides additional therapeutic options, including pharmacological interventions (e.g., GLP-1 receptor agonists such as semaglutide, or agents like bupropion for patients with emotional eating patterns) and bariatric surgery for eligible individuals [7,15]. Interdisciplinary care involving physicians, dietitians, physiotherapists, and psychotherapists is recommended to optimize weight loss and long-term adherence. Importantly, weight reduction not only lowers baseline risk but may also enhance the effectiveness of antibiotic prophylaxis [7]. Lymphedema is another major predictor of recurrence. Conservative management of lymphedema can reduce recurrence risk and includes manual lymphatic drainage, elevation of the affected limb, muscle-pumping exercises, use of compression garments such as multilayer compression stockings, and appropriate skin care [16]. Graduated compression stockings (GCS) are frequently prescribed; they exert the greatest pressure at the ankle with gradually decreasing compression proximally. Class 1 stockings provide <20 mmHg, Class 2 around 20–30 mmHg, and Class 3 ≥30 mmHg [7]. However, compliance with GCS can be challenging—non-adherence rates of up to 60% have been reported due to discomfort, skin irritation, or cosmetic concerns [7]. Moreover, they are contraindicated in patients with significant peripheral arterial disease, neuropathy, or material allergy. Despite these limitations, clinical studies have confirmed their benefit: in a randomized single-center trial, compression therapy reduced recurrence risk with a hazard ratio (HR) of 0.23 (95% CI 0.09–0.59, *p* = 0.002), provided patients adhered to wearing them most days of the week [7]. In a small single-center nonblinded trial involving patients with chronic leg edema and cellulitis, compression therapy was associated with a lower recurrence rate compared to patient education alone (15% vs. 40%) [17]. In cases related to chronic venous insufficiency, where conservative measures fail, further evaluation with intravascular ultrasound and venous stenting may be considered to improve venous outflow and reduce recurrence risk [7]. If conservative lymphedema therapy is insufficient, surgical procedures such as excision or liposuction of fibroadipose tissue or lymphovenous bypass may be considered [18]. Other anatomical abnormalities of the lymphatic system may also predispose patients to recurrence. Importantly, a previous episode of erysipelas itself remains one of the strongest predictors of future recurrence, which is a non-modifiable factor [2]. In women after mastectomy with lymph node dissection, prevention can be more complex. While lymphedema in such cases is not reversible, preventive measures include careful skin protection and avoiding blood draws or blood pressure measurements in the affected arm. Other chronic diseases also play a role.Chronic obstructive pulmonary disease (COPD) has been linked to increased erysipelas recurrence [2], highlighting the importance of smoking cessation, which additionally improves microcirculation and tissue healing [7]. Smoking cessation strategies include behavioral interventions and pharmacological support, such as nicotine replacement therapy, bupropion, or cytisine [15]. Skin integrity and hygiene are additional key factors. Poor hygiene, interdigital mycoses, or chronic eczema significantly increase the risk of recurrence by providing bacterial entry points. Regular moisturizing, treatment of abrasions or fissures, and targeted antifungal therapy (topical or systemic) for tinea pedis and onychomycosis are essential [7,19]. Preventive measures also include appropriate footwear, avoidance of barefoot walking in public places, and maintaining proper foot and leg hygiene. Prolonged immobilization in patients with venous insufficiency or lymphedema may further increase recurrence risk. These interventions, when applied consistently, have the potential to substantially reduce recurrence rates and may decrease the need for antibiotic prophylaxis. At present, no vaccines are available against erysipelas, and prior infection does not confer lasting immunity. Ultimately, non-pharmacological strategies should be regarded as the cornerstone of recurrence prevention. As discussed later in this review, antibiotic chemoprophylaxis, although effective, carries the risk of adverse effects, microbiota disruption, and antimicrobial resistance. Therefore, prioritizing lifestyle interventions, lymphedema management, and optimized control of comorbidities is essential. Obesity, in particular, not only increases the baseline risk of recurrence but also reduces the success rate of antibiotic prophylaxis, further underscoring the need for comprehensive, multidisciplinary approaches that target modifiable risk factors as a first-line strategy. Non-pharmacological strategies for prevention of recurrent erysipelas are summarized in Table 3.

## 5. Chemoprophylaxis of Recurrent Erysipelas—Available Treatment Modalities

Recurrent erysipelas is most frequently caused by group B, G, or C streptococci, and less commonly by group A streptococci (GAS) [8]. Chemoprophylaxis refers to the prevention of disease through pharmacological treatment. While antibiotic prophylaxis for recurrent erysipelas is available, it is not without limitations. Long-term antibiotic therapy is associated with adverse effects, drug–drug interactions (particularly relevant in patients with multiple comorbidities requiring polypharmacy), and disruption of the normal gut microbiota. Therefore, the decision to initiate chemoprophylaxis must be individualized, carefully weighing the potential benefits against the associated risks. Prophylaxis should not be applied routinely, but only after a thorough clinical assessment. Relevant considerations include the severity and frequency of prior episodes, the risk of complications, underlying conditions that predispose to recurrence (such as lymphedema) and their modifiability, as well as the potential for antimicrobial resistance during prolonged use. Importantly, non-pharmacological interventions should always be prioritized and implemented first [6,7]. Chronic conditions such as lymphedema and obesity are the principal risk factors for recurrent erysipelas. These patients are the main candidates for chemoprophylaxis. In contrast, acute risk factors such as minor local trauma typically predispose to the initial episode of erysipelas rather than to recurrence. Consequently, not all patients experiencing a first episode are at significant risk of relapse, and chemoprophylaxis should be reserved for those with a clearly elevated risk [2]. Antibiotics used for chemoprophylaxis may be administered orally or via intramuscular injection. When selecting the appropriate agent, recent microbiological results should be considered, and the same antibiotic should not be used for both treatment of the acute infection and prophylaxis. Patient-specific factors such as hepatic or renal impairment, pregnancy, and age must also be taken into account due to the risk of hepatotoxicity, altered pharmacokinetics, and the need for dose adjustments [6]. Importantly, current recommendations emphasize that antibiotic prophylaxis is indicated only for non-purulent infections, directed primarily against β-hemolytic streptococci, particularly *Streptococcus pyogenes* (GAS) [7]. The most widely used intramuscular option is benzathine penicillin G (also known as benzathine benzylpenicillin, or trade name in some countries “debecillin”; BPG). The recommended dose is 1.2 to 2.4 million units intramuscularly, typically administered once every 4 weeks (±1 week). In clinical practice, prophylaxis is usually initiated with 4-weekly injections; however, in patients who experience breakthrough recurrences despite this regimen, the dosing interval may be shortened to every 2–3 weeks. Some sources also describe administration every 2 weeks in selected high-risk cases. This flexibility allows for individualized tailoring of prophylaxis depending on the recurrence pattern and patient response [6,7,8]. Oral prophylactic regimens include phenoxymethylpenicillin (penicillin V) 250 mg twice daily, erythromycin 250–500 mg once daily, azithromycin 250 mg once daily, or clarithromycin 500 mg once daily. In patients intolerant to penicillin, cephalexin 125–250 mg twice daily or cefadroxil may be used. Most guidelines identify penicillin V as the first-line choice, with erythromycin as the preferred option for penicillin-allergic patients [6,7,8]. In obese patients, higher doses may be required; for example, individuals with BMI ≥ 33 kg/m^2^ are advised to receive penicillin V 500 mg twice daily instead of 250 mg twice daily, and case reports describe even higher doses (up to 2 g twice daily). Similarly, in patients weighing >100 kg, guidelines recommend doubling the standard dose of prophylactic antibiotics [7]. While lower doses are generally well tolerated, higher doses in obese patients have been associated with increased gastrointestinal side effects [7]. Alternative regimens should be considered in cases of allergy or intolerance. For patients with non-severe hypersensitivity to penicillin, cephalexin or cefadroxil may be used. In severe penicillin allergy, macrolides remain the preferred option, with erythromycin being the most widely studied [7]. If first-line prophylaxis fails, some guidelines suggest clindamycin 150 mg once daily, cephalexin 125 mg once daily, doxycycline 50 mg once daily, or trimethoprim–sulfamethoxazole (TMP-SMX) in low prophylactic doses [7]. Several in vitro and clinical studies support doxycycline’s activity against both *Staphylococcus aureus* and *Streptococcus pyogenes* (GAS), which justifies its consideration as an alternative prophylactic option [20,21]. TMP-SMX has also demonstrated efficacy in treating skin and soft tissue infections caused by GAS, including cellulitis and impetigo [22], suggesting a theoretical role in prophylaxis. However, no standardized prophylactic regimens with TMP-SMX have been established, and current evidence remains scarce [7]. A key practical distinction between the available regimens lies in treatment adherence. Oral prophylactic agents must be taken daily, often twice daily, which makes patient compliance a critical factor in their effectiveness. Missed doses significantly reduce prophylactic efficacy. Moreover, gastrointestinal disturbances such as vomiting or diarrhea, as well as conditions associated with malabsorption or gastrointestinal intolerance, can impair drug absorption and thereby compromise the prophylactic effect. In contrast, intramuscular BPG offers a clear advantage: a single injection administered approximately every four weeks provides reliable systemic levels independent of gastrointestinal function and removes the burden of daily adherence. For many patients, this schedule is more convenient and ensures more consistent prophylaxis [6]. It is also important to note that penicillin allergy is frequently overreported, and many patients who report such an allergy can actually tolerate β-lactam antibiotics after proper evaluation [23,24]. This has important clinical implications, as penicillin remains the most effective and evidence-based prophylactic option. Antibiotic chemoprophylaxis regimensfor recurrent erysipelas are summarized in Table 4.

## 6. Efficacy of Antibiotic Prophylaxis in Recurrent Erysipelas

Evidence from randomized controlled trials and systematic reviews demonstrates that antibiotic prophylaxis can meaningfully reduce the risk of recurrent cellulitis or erysipelas. A Cochrane meta-analysis of five RCTs (Randomized Controlled Trials) involving adults (mostly aged 46–70 years) with one or two prior episodes of cellulitis or erysipelas showed that either intramuscular or oral penicillin, or oral erythromycin, significantly lowered the likelihood of recurrence compared with placebo or no treatment. Depending on study design, prior episodes had occurred within the previous three months to three years. Across included trials, prophylaxis also reduced the incidence rate (episodes per person-month) and prolonged the time to the next recurrence. However, prophylaxis had no significant effect on mortality or hospitalization rates [6,25]. The largest single study, PATCH I, conducted by the British Association of Dermatologists, randomized 274 patients with ≥2 previous episodes within three years. Daily oral penicillin V (250 mg twice daily) administered for 12 months lowered the risk of recurrence by 45% compared to placebo. Interestingly, the benefit was not observed in participants with chronic edema, BMI ≥ 33 kg/m^2^, or those with ≥3 prior episodes, suggesting that persistent risk factors may blunt the effect of antibiotics and should be optimized alongside prophylaxis [7]. Other studies have assessed intramuscular regimens. In one trial, BPG 1.2 million units every two weeks prevented all recurrences at one-year follow-up (0/24 vs. 26% in controls). Observational data also support this approach: in a retrospective series, monthly injections (2.4 million units every three weeks) were associated with a significantly lower incidence of cellulitis (0.73 vs. 1.25 episodes per patient-year, *p* < 0.001). Nevertheless, these findings are limited by small sample sizes, single-center designs, and lack of randomization [7]. Macrolides have also been tested. In an open-label RCT of 32 patients with ≥2 episodes in the previous year, none of the 16 erythromycin recipients (250 mg twice daily for 18 months) relapsed, compared to 50% of controls (8/16, *p* < 0.001). Gastrointestinal side effects occurred in 20% of participants, necessitating a switch to penicillin V. Although encouraging, the small scale and non-blinded design of this study limit generalizability [7]. Taken together, the evidence indicates that antibiotic prophylaxis—most commonly oral or intramuscular penicillin, with erythromycin as an alternative—can substantially reduce recurrence risk in patients with recurrent cellulitis or erysipelas, though efficacy may be diminished in those with persistent predisposing conditions such as chronic lymphedema or severe obesity [6,7]. Overall, current evidence suggests that antibiotic prophylaxis is effective in reducing recurrent episodes of erysipelas or cellulitis, particularly in selected patient groups. However, the available data are limited, with relatively small sample sizes and heterogeneous study designs, and the benefit may be attenuated in those with persistent risk factors such as chronic lymphedema. Importantly, concerns remain regarding the development of antimicrobial resistance; for instance, resistance to erythromycin has been increasingly reported in *Streptococcus pyogenes*, and is observed in up to 30–45% of groups B, C and G streptococci [7].

## 7. Duration of Chemoprophylaxis for Recurrent Erysipelas

Patients receiving antibiotic prophylaxis for recurrent erysipelas should remain under regular medical supervision. Follow-up visits should assess the effectiveness of prophylaxis, treatment tolerability, the occurrence of adverse effects, and potential drug–drug interactions that may require modification of therapy. Such reviews are recommended at least every six months and should evaluate the success of prophylaxis, and consider whether to continue, discontinue, or change the regimen, taking into account patient preference and the risk of antimicrobial resistance [6]. Currently, there are no universally established guidelines on the optimal duration of chemoprophylaxis. Most studies support continuation for 6–12 months, with some recommending up to 18 months [7,26]. In general, long-term low-dose prophylactic antibiotics are well tolerated. Importantly, once prophylaxis is discontinued, the protective effect is lost, which explains why longer courses are more effective. Extension of therapy is often advised, particularly if relapse occurs after discontinuation, or until modifiable predisposing factors are corrected. In certain patients, lifelong prophylaxis may be necessary when risk factors cannot be adequately reduced or eliminated, for example, in severe obesity or persistent lymphedema. According to the BLS, penicillin V 250 mg twice daily (500 mg twice daily if BMI ≥ 33 kg/m^2^) is recommended for one year, followed by 250 mg once daily for another year; if recurrence occurs after this period, lifelong prophylaxis is suggested [7,27]. Further studies are needed to determine the optimal duration of therapy for different regimens. For instance, erythromycin prophylaxis for 18 months was effective in preventing recurrence, while six months of low-dose penicillin produced only a modest and statistically insignificant reduction in relapse rates [26,27]. Trials of intramuscular BPG in patients with chronic lymphedema of the upper and lower limbs, given every 14–21 days for several years, showed excellent outcomes, with a reduction in recurrence risk of up to 95%, good tolerability, and no clinical evidence of resistance [28]. Chemoprophylaxis should be stopped or switched if erysipelas or cellulitis occurs despite ongoing treatment [6]. Importantly, prophylaxis is intended only for patients without active infection, as its purpose is to prevent recurrence after the resolution of an acute episode in individuals at high risk of relapse. If symptoms of an acute infection appear (e.g., fever, erythema, pain), prophylaxis must be interrupted and full-dose antibiotic therapy initiated for the acute episode. Once symptoms resolve, prophylaxis may be restarted if indicated, potentially with an alternative regimen if recurrence occurred during prophylaxis. In uncertain cases, laboratory markers such as CRP (C-reactive protein) or leukocyte count with neutrophilia may help confirm acute infection before resuming prophylaxis. The decision to initiate prophylaxis should also be based on recurrence risk. Available data emphasize that correction of modifiable factors such as obesity and lymphedema should be the first step. Prophylaxis is not recommended after a single episode of erysipelas or cellulitis, but rather in cases of recurrence. Recommendations differ: some guidelines allow initiation after two episodes within 12 months, whereas others advocate a stricter threshold of three to four episodes per year despite risk factor modification [29]. The PATCH II trial even included patients after their first episode, but the observed reduction in recurrence did not reach statistical significance, leaving the evidence insufficient for definitive clinical recommendations [30]. In patients who continue to experience recurrences despite prophylaxis, several aspects should be carefully reassessed. First, treatment adherence must be verified: whether the patient is taking the medication daily as prescribed, or, in the case of intramuscular BPG, attending scheduled injections. It is also important to confirm that the prescribed dose is adequate, since patients with a BMI ≥ 33 kg/m^2^ should receive higher doses of oral penicillin V; the injection interval may need to be shortened to every 2–3 weeks instead of every 4 weeks in cases of breakthrough infections [6,7]. The possibility of antimicrobial resistance must also be considered; for instance, erythromycin resistance is increasingly reported and may necessitate switching to penicillin in the absence of contraindications [7]. Another consideration is the duration of prophylaxis, as shorter courses may be insufficient and the protective effect is lost upon discontinuation, meaning that some patients require lifelong prophylaxis. Finally, attention should be given to the adequacy of risk factor management. Conditions such as chronic lymphedema or severe obesity not only predispose to recurrence but may also diminish the efficacy of chemoprophylaxis if not sufficiently controlled [2,7]. A summary of key clinical studies and systematic reviews evaluating antibiotic prophylaxis in recurrent cellulitis/erysipelasis presented in Table 5.

## 8. Chemoprophylaxis of Recurrent Erysipelas—Adverse Effects and Complications

It is important to emphasize that prolonged antibiotic therapy, in some cases extending to lifelong administration, carries a risk of adverse effects, complications, and limitations [7]. Chronic antibiotic use, particularly oral prophylaxis, disrupts the intestinal microbiota and predisposes to CDI. This may lead to dehydration, fluid and electrolyte imbalance, and in severe cases, complications such as intestinal obstruction, toxic megacolon, or even death. The risk of CDI is highest with clindamycin, moderate with macrolides and penicillins, and relatively low with tetracyclines (e.g., doxycycline, which is rarely used for prophylaxis of erysipelas) [32]. Obese patients often require higher or even doubled doses of oral antibiotics to achieve adequate prophylactic efficacy. However, higher doses are frequently associated with gastrointestinal intolerance, including nausea, diarrhea, or abdominal discomfort, which can limit adherence and overall effectiveness [7]. As with any drug, antibiotic use is associated with potential side effects and contraindications. Hypersensitivity reactions and antibiotic-induced liver injury may occur, sometimes independently of dose. Adverse events may be common, uncommon, or rare, depending on the agent. For example, erythromycin has been associated with cardiac arrhythmias such as ventricular tachycardia or torsades de pointes, as well as agranulocytosis [33]. Azithromycin can cause gastrointestinal upset, rash, photosensitivity, transient cholestatic jaundice, seizures, anxiety, and thrombocytopenia [34]. Clarithromycin may lead to candidiasis, vaginal infections, leukopenia, and QT interval prolongation on electrocardiogram (ECG). In fact, macrolide therapy, including clarithromycin, has been linked to delayed cardiac repolarization and torsades de pointes. Therefore, clarithromycin should not be prescribed to patients with congenital or documented acquired QT interval prolongation, a history of ventricular arrhythmia, or hypokalemia [35]. Doxycycline, although rarely used in erysipelas prophylaxis, is contraindicated in pregnancy and may cause phototoxic reactions [36]. Beyond individual drug toxicities, antimicrobial resistance is a growing limitation of long-term prophylaxis. While *Streptococcus pyogenes* remains universally susceptible to penicillin, resistance to macrolides has risen considerably in many regions. Recent European surveillance data indicate variable rates, ranging from <5% in some northern countries to 20–40% in parts of southern Europe [37,38]. In Spain, retrospective analyses of invasive isolates between 2007 and 2020 documented persistent macrolide resistance around 8–9% and clindamycin resistance of ~4% [38]. In contrast, other regional studies reported substantially higher resistance levels, exceeding 30% in certain countries such as Türkiye and Belgium [39]. In pediatric populations, resistance rates may be particularly elevated: for instance, in children with acute otitis media, macrolide resistance of S. pyogenes reached 32% in Bulgarian isolates [40]. The underlying mechanisms include ribosomal methylases (e.g., erm genes) and active efflux pumps (e.g., mef(A/E)–mrs(D)), which are unevenly distributed across regions and frequently associated with mobile genetic elements and clonal lineages [37]. Consequently, resistance levels vary not only with bacterial genetics but also with local prescribing practices and antibiotic pressure. Such variability highlights the need to consider local resistance data when selecting prophylactic regimens. The ecological consequences of long-term antibiotic use must also be taken into account. Prophylaxis exerts selective pressure that may facilitate colonization with multidrug-resistant organisms, including MRSA and resistant Enterobacteriaceae. While penicillin prophylaxis itself has not been directly linked to MRSA emergence, altering the skin and mucosal microbiota through chronic antibiotic exposure can create ecological niches for resistant pathogens. This reinforces the importance of using antibiotic prophylaxis judiciously, restricting it to patients with frequent recurrences despite optimal management of risk factors, and reassessing the indication regularly. Another clinically important issue is penicillin allergy. Self-reported allergy is common, but evidence shows that up to 90% of such patients can actually tolerate β-lactam antibiotics after proper evaluation [23,24]. Careful allergy history, and if necessary, skin testing or oral challenge, should therefore precede the use of alternative agents. This is particularly relevant in recurrent erysipelas, where penicillin remains the most effective and evidence-based prophylactic option. Patients with confirmed non-severe hypersensitivity may still receive cephalosporins, whereas those with severe immediate hypersensitivity should be switched to macrolides, doxycycline, or other alternatives [7]. However, the rising prevalence of macrolide resistance in *S. pyogenes* further limits the utility of these agents, strengthening the argument for confirming penicillin allergy whenever possible. Drug–drug interactions should always be considered, as patients eligible for prophylaxis often have comorbidities requiring multiple medications. For instance, erythromycin may moderately interact with apixaban, leading to increased apixaban levels and a higher risk of bleeding. A similar interaction exists with rivaroxaban [33]. Intramuscular BPG administered in patients receiving therapeutic anticoagulation with NOACs (Novel Oral Anticoagulants) or low molecular weight heparin carries a risk of hematoma, sometimes extensive. This is not an absolute contraindication but requires special caution. If possible, the intramuscular route should be avoided in favor of oral alternatives. If intramuscular injection is necessary, the risk–benefit ratio should be carefully evaluated, and measures such as deep injection into a large muscle and firm post-injection compression should be applied. Oral alternatives are generally more practical in these patients. However, some studies have suggested that BPG given to anticoagulated patients did not result in significant bleeding complications on the day of injection, within seven days post-injection, or during hospitalization [41]. In summary, while antibiotic prophylaxis can be effective in preventing recurrent erysipelas, its long-term use is limited by adverse drug effects, interactions, macrolide resistance, and the frequent overreporting of penicillin allergy. Careful patient selection, monitoring, and management of modifiable risk factors remain essential to maximize efficacy while minimizing harm. Limitations, challenges and adverse effects of antibiotic prophylaxis in recurrent erysipelas are summarized in Table 6 and Table 7.

## 9. Discussion

Recurrent erysipelas represents a significant clinical challenge due to its high rate of relapse, particularly among patients with persistent predisposing factors such as chronic lymphedema, obesity, and venous insufficiency. As highlighted in previous sections, recurrence rates range from 11% in outpatients during the first year to as high as 46% of hospitalized patients within three years [2,4]. These figures emphasize the considerable burden of this condition on both patients and healthcare systems. A major issue in the management of recurrent erysipelas is the bidirectional relationship with lymphedema. Each episode of erysipelas may further damage lymphatic vessels, thereby worsening lymphedema, which in turn increases the risk of subsequent infections [8,11]. This vicious cycle complicates prevention and explains why most recurrences occur in the same anatomical location as the initial episode [4,13]. Effective prevention strategies must therefore target not only acute infection but also underlying risk factors. Non-pharmacological strategies are essential and should always precede antibiotic prophylaxis. Measures such as weight reduction, strict glycemic control in diabetic patients, smoking cessation, and meticulous skin care have the potential to lower recurrence risk [15,19]. In addition, lymphedema management with compression therapy, limb elevation, and physiotherapy has shown measurable benefits, with clinical trials demonstrating a reduction in recurrence rates [17]. Nevertheless, these interventions require long-term adherence and patient motivation, which may limit their effectiveness in routine clinical practice. Antibiotic prophylaxis remains the most widely studied and effective intervention in preventing recurrence. Evidence from randomized controlled trials and observational studies supports its efficacy. In the PATCH I trial, penicillin V 250 mg twice daily for 12 months reduced the risk of recurrence by approximately 45% compared with placebo (22% vs. 37% recurrence over 3 years; HR 0.55, 95% CI 0.35–0.86) [31]. Similarly, Kremer et al. demonstrated that prolonged prophylaxis with oral erythromycin 250 mg twice daily for 18 months was highly effective, as none of the patients in the prophylaxis group developed recurrent infections, compared with 50% relapse in the control group [26]. A Cochrane review confirmed these observations, concluding that prophylactic antibiotics reduce recurrence during active therapy, but long-term benefits, cost-effectiveness, and the risk of resistance remain uncertain [25]. Notably, the protective effect diminishes after discontinuation of antibiotics, as shown in both PATCH I and earlier studies such as PATCH II [30]. The optimal duration of chemoprophylaxis therefore remains unclear. Current evidence supports treatment for 6—18 months, although some guidelines recommend lifelong prophylaxis in individuals with unmodifiable high-risk conditions such as chronic lymphedema [7,27]. Importantly, once prophylaxis is discontinued, the protective effect is rapidly lost, highlighting the need for individualized treatment duration based on risk factor modification and patient-specific circumstances. A key practical distinction between available regimens lies in adherence. Oral prophylactic agents must be taken daily, often twice daily, which makes compliance a critical determinant of effectiveness. Missed doses significantly reduce prophylactic efficacy, and gastrointestinal disturbances (e.g., vomiting, diarrhea) or malabsorption further compromise outcomes. In contrast, intramuscular BPG provides reliable systemic levels independent of gastrointestinal absorption and eliminates the burden of daily adherence. For many patients, a 4-weekly injection is more convenient and ensures more consistent prophylaxis [6]. However, in anticoagulated patients, intramuscular injections carry a risk of hematoma formation. Although not an absolute contraindication, they require careful risk–benefit assessment, cautious injection technique, and monitoring [41]. Adverse effects, antimicrobial resistance, and drug–drug interactions are key limitations to long-term prophylaxis. The risk of CDI, hepatotoxicity, cardiac arrhythmias, and hematological abnormalities has been documented with various agents [32,33,34,35,36]. Obese patients, who often require higher or doubled oral doses for efficacy, may be particularly prone to gastrointestinal side effects, further complicating adherence. Beyond individual toxicities, antimicrobial resistance is a growing limitation. While *Streptococcus pyogenes* (GAS) remains universally susceptible to penicillin, resistance to macrolides has risen considerably in many regions. Recent European surveillance data indicate variable rates, ranging from <5% in some northern countries to 20–40% in parts of southern Europe [37,38]. In Spain, retrospective analyses of invasive isolates between 2007 and 2020 documented persistent macrolide resistance around 8–9% and clindamycin resistance of ~4% [38], while other regional studies reported even higher rates (>30%), particularly in Türkiye [39]. In pediatric isolates, resistance rates may be elevated, with up to 32% macrolide resistance observed in otitis media strains [40]. The underlying mechanisms include ribosomal methylases (erm genes) and active efflux pumps (mef(A/E)–mrs(D)), which are unevenly distributed across regions and often linked to mobile genetic elements and clonal lineages [37]. Such variability highlights the need to consider local resistance data when selecting prophylactic regimens. Long-term antibiotic exposure also exerts selective pressure that may facilitate colonization with multidrug-resistant organisms, including MRSA and resistant Enterobacteriaceae. While penicillin prophylaxis itself has not been directly linked to MRSA emergence, chronic antibiotic use alters the skin and gut microbiota, potentially creating ecological niches for resistant pathogens. This reinforces the importance of restricting chemoprophylaxis to patients with frequent recurrences despite optimal risk-factor management, and of reassessing the indication regularly, as emphasized by current guidelines (e.g., IDSA, BLS) [29,42]. Another important limitation is the lack of standardization across the literature and clinical practice. Even the very definition of recurrent erysipelas remains inconsistent, with some sources requiring ≥2 episodes within 12 months, others ≥2 episodes over three years, and still others restricting recurrence to the identical anatomical site. Similarly, there is no consensus regarding the threshold for initiating chemoprophylaxis, nor the recommended duration of therapy. Data on certain agents are particularly scarce—for example, trimethoprim–sulfamethoxazole has been mentioned as a potential prophylactic option, but no standardized dosing regimens exist outside treatment settings. These uncertainties are clinically relevant, given that the populations most affected—patients with obesity, chronic lymphedema, or cancer survivors after lymph node dissection—are also those at highest risk of recurrence and prophylaxis failure.

## 10. Conclusions

Recurrent erysipelas is a complex condition that requires a multifaceted approach. While antibiotic prophylaxis significantly reduces recurrence risk, its success is closely linked to parallel management of underlying risk factors. Non-pharmacological interventions, particularly weight reduction, metabolic control, smoking cessation, and lymphedema management, remain essential and in some patients may be sufficient to prevent recurrence. Long-term antibiotic prophylaxis, although effective, carries risks that must be carefully balanced against its benefits. Importantly, the lack of standardized recommendations regarding the initiation, choice, and duration of prophylaxis underscores the need for further research. Future directions should focus not only on optimizing antimicrobial strategies but also on advancing non-antibiotic approaches, including lifestyle-based prevention and the development of vaccines against *Streptococcus pyogenes*. The development of a vaccine against group A streptococcus (GAS) remains a major scientific challenge. The pathogen expresses multiple virulence factors and exhibits wide strain diversity that varies geographically, making it difficult to design a broadly protective formulation. Early attempts, including a non-randomized trial with 100 participants who received a vaccine composed of heat-inactivated preparations from 12 streptococcal serotypes, suggested a possible reduction in the frequency of recurrent cellulitis. More recent work has focused on molecular vaccine candidates. Among them, the J8-DT/HD-MAP construct has shown promising results in preclinical models, significantly lowering GAS colonization in animals. To date, however, no large-scale human clinical trials have been completed, and further research is necessary before streptococcal vaccination can be considered a realistic preventive option for recurrent erysipelas [7].

## 11. Broader Implications and Future Directions

Recurrent erysipelas has a profound impact not only on clinical outcomes but also on patients’ quality of life. Repeated hospitalizations, recurrent pain, limited mobility, and prolonged work absence contribute to a considerable psychosocial and socioeconomic burden. From a health system perspective, the prevention of recurrence can reduce hospital admissions and costs, underscoring the importance of effective preventive strategies. Despite encouraging data from randomized trials, most available studies are limited by small sample sizes, short follow-up, and heterogeneous inclusion criteria. Large, multicenter randomized controlled trials are needed to determine the optimal antibiotic regimen, treatment duration, and the subgroups most likely to benefit. Another key challenge is the absence of consensus regarding the definition of recurrent erysipelas itself, including whether recurrence must occur in the same anatomical region.

Equally important is the broader context of lifestyle medicine and holistic patient care. Since obesity is one of the strongest risk factors for recurrence, prevention and treatment strategies must address it comprehensively. The emerging field of obesity medicine emphasizes an interdisciplinary approach that combines medical specialists (infectious diseases, cardiology, endocrinology, orthopedics) with allied professionals such as dietitians, psychotherapists, and physiotherapists. Both pharmacological options (e.g., GLP-1 receptor agonists such as semaglutide for patients with increased appetite and delayed satiety; bupropion for individuals with emotional eating or stress-related overeating) and bariatric surgery may play important roles, depending on patients’ obesity phenotype [15].

It is also important to recognize that one of the major reasons for prophylaxis failure is the persistence of strong risk factors, particularly obesity. Several studies have shown that patients with severe obesity or chronic edema may continue to relapse despite receiving long-term antibiotic prophylaxis. In these cases, the effectiveness of chemoprophylaxis appears attenuated, underscoring the critical need to address modifiable conditions in parallel. Weight reduction—achieved through lifestyle interventions, pharmacological therapy, or bariatric surgery—may therefore not only reduce the baseline risk of recurrence but also improve the long-term success of antibiotic prophylaxis.

From the patient’s perspective, intramuscular BPG administered every 3–4 weeks is often the most convenient option, as it avoids the need for daily oral intake and issues with gastrointestinal absorption or adherence. In some cases, however, more frequent injections (every 2–3 weeks) are required to maintain efficacy. An important consideration is that an increasing proportion of patients—particularly those with obesity-related comorbidities such as atrial fibrillation—are chronically treated with therapeutic anticoagulation. In this setting, intramuscular injections may raise concerns regarding the risk of hematoma, although evidence suggests that the absolute risk is not uniformly high. Nevertheless, this potential complication needs to be weighed carefully against the advantages of simplified dosing and improved compliance associated with injectable prophylaxis.

Similarly, the control of other chronic diseases is essential. Optimized glycemic control in diabetes reduces impaired wound healing and lowers the risk of infection, while smoking cessation improves microcirculation and tissue repair. Smoking cessation itself may require combined behavioral and pharmacological interventions, including bupropion or cytisine. Such holistic strategies highlight the importance of integrating lifestyle medicine into the management of recurrent erysipelas, shifting the focus from treatment to prevention. On the population level, public health measures and health education aimed at reducing obesity and other lifestyle-related diseases are equally crucial.

Finally, novel approaches are under investigation. Vaccine development against group A streptococcus (GAS) remains challenging due to the pathogen’s antigenic diversity and geographic variability. A non-randomized trial using a heat-inactivated streptococcal vaccine suggested potential benefit in reducing recurrence, and more recent animal studies of the J8-DT/HD-MAP vaccine demonstrated significant reductions in *S. pyogenes* colonization. However, no large clinical trials in humans are yet available [7].

Taken together, recurrent erysipelas should be viewed as a condition at the intersection of infectious diseases, chronic disease management, and lifestyle medicine. Its prevention requires an integrated, multidisciplinary approach that balances antimicrobial prophylaxis with risk factor modification, holistic care, and public health interventions.

## Figures and Tables

**Table 1:** Definitions of recurrent erysipelas across guidelines and studies.

Source/Guideline	Definition of Recurrence	Notes
NICE (2019) [6]	≥2 distinct, documented episodes within 12 months	Applies to cellulitis and erysipelas; triggers consideration of prophylaxis [6]
Australian Lymphology Association (2015) [7]	≥2 episodes per year despite skin care and treatment of risk factors	Focuses on chronic edema/lymphedema patients [7]
BLS (2016) [7]	≥2 episodes per year	Similar to Australian guideline [7]
IDSA (2014) [7]	3–4 episodes per year despite control of risk factors	Stricter threshold [7]
South Korean SSTI guideline (2017) [7]	3–4 episodes per year	Similar to IDSA [7]
Sanford Guide (2025) [8]	≥2 episodes within 3 years	Less strict; “frequent episodes” definition [8]
Inghammar et al. (2014) [4]	>1 episode, irrespective of time interval	No fixed temporal criterion [4]
Li et al. (2021, China) [9]	Recurrence defined only if in the same anatomical location	Excludes new episodes at different sites [9]
Matych et al. (2025, Poland) [10]	Recurrent erysipelas accounted for 75.7% of cases	No time-based threshold provided [10]

**Table 2:** Risk factors of recurrent erysipelas.

Type of Risk Factor	Risk Factor	Pathomechanism
non-modifiable	age	Age-related skin changes.
	sex	Physique, hormonal and differences.
	history of previous erysipelas episodes	Recovery from erysipelas does not confer immunity, and the risk of complications can predispose individuals to recurrent episodes.
modifiable	lymphedema	Impairs lymphatic function and tissue viability, increasing the risk of infection.
	obesity	Adverse effects on lymphatic function and lymph node architecture, increasing susceptibility to skin infections.
	diabetes	Suboptimal glycemic control increases the risk of infection, diabetic complications lead to ulcer formation.
	venous insufficiency	Sustained venous hypertension leads to chronic inflammatory changes and edema.
	cancer	Edema resulting from tumor invasion, lymph node resection, or radiation therapy increases the risk of infection.
	immunodeficiency	Weakened immune defenses. Increases the risk of infection.
	skin trauma	Provides a portal of entry for bacteria.
	dermatological conditions	Harboring pathogenic bacteria, including group A streptococcus.

**Table 3:** Non-pharmacological strategies for prevention of recurrent erysipelas: benefits and limitations.

Intervention	Potential Benefits	Limitations/Challenges
Weight reduction(diet, physical activity, bariatric or pharmacological treatment)	Lowers recurrence risk, improves metabolic profile, enhances success of antibiotic prophylaxis	Requires long-term adherence; weight loss is difficult to sustain; variable access to obesity treatments
Glycemic control in diabetes	Improves wound healing, reduces infection portals, lowers recurrence risk	Demands strict glucose monitoring; suboptimal control remains common
Smoking cessation	Improves microcirculation, reduces chronic inflammation, lowers risk of COPD and delayshealing	High relapse rates; requires behavioral and/or pharmacological support
Lymphedema management(compression, manual drainage, physiotherapy, skin care)	Reduces recurrence (HR ~0.23 in RCT with compression therapy); improves quality of life	Compliance often poor (<50%); discomfort, skin irritation, cosmetic concerns
Treatment of fungal infections(tinea pedis, onychomycosis)	Eliminates common entry portals; reduces recurrence	Risk of reinfection; requires long-term topical or systemic therapy
Venous insufficiency treatment(conservative or stenting)	May reduce edema and recurrence in selected cases	Limited availability; requires specialized expertise
General skin care&hygiene(moisturization, footwear, wound protection)	Reduces microfissures and entry points for pathogens	Requiresongoingpatient education and daily effort

**Table 4:** Antibiotic chemoprophylaxis regimens for recurrent erysipelas.

Route	Antibiotic	Standard Dose	Alternative/High-Risk Dosing	Notes	Sources
Intramuscular	Benzathinepenicillin G (debecillin)	1.2–2.4 million units every 4 weeks (±1 week)	Interval shortened to every 2–3 weeks in patients with breakthrough recurrences	First-line; independent of GI absorption; convenient monthly dosing	[6,7,8]
Oral	Penicillin V (phenoxymethylpenicillin)	250 mg twice daily	500 mg twice daily if BMI ≥ 33; case reports of up to 2 g twice daily; double dose if >100 kg	First-line oral prophylaxis	
	Erythromycin	250–500 mg once daily	—	Preferred in penicillin-allergic patients	
	Azithromycin	250 mg once daily	—	Option in penicillin allergy	
	Clarithromycin	500 mg once daily	—	Option in penicillin allergy	
	Cephalexin	125–250 mg twice daily	125 mg once daily (alternative regimen)	For non-severe penicillin allergy	
	Cefadroxil	Not standardized	—	For non-severe penicillin allergy	
	Clindamycin	150 mg once daily	—	Considered if first-line fails	[7]
	Doxycycline	50 mg once daily	—	Active vs *S. aureus* & GAS; option if intolerance to other agents	[7,20,21]
	TMP-SMX	Low prophylactic doses (not standardized)	—	Efficacy in GAS infections; theoretical prophylaxis role but limited evidence	[7,22]

**Table 5:** Summary of key clinical studies and systematic reviews evaluating antibiotic prophylaxis in recurrent cellulitis/erysipelas. Evidence consistently shows that prophylaxis with oral or intramuscular antibiotics reduces recurrence during active treatment, although protection diminishes after discontinuation. The strength of evidence varies: PATCH I provides the most robust RCT data, whereas Kremer’s trial and observational studies are limited by small sample sizes and open-label design. The Cochrane review confirms overall efficacy but highlights uncertainties regarding optimal duration, cost-effectiveness, and long-term safety.

Study/Source	Population	Intervention	Duration	Main Results	Notes
PATCH II (Thomas et al., 2012, Br J Dermatol) *n* = 123 [30]	Patients with ≥1 previous episode of leg cellulitis	Penicillin V 250 mg BID (vs. placebo)	6 months	Recurrence: 20% vs. 33% (HR 0.53, 95% CI 0.26–1.07, *p* = 0.08)	Trend toward benefit; effect only during prophylaxis; underpowered
PATCH I (Thomas et al., 2013, NEJM) *n* = 274 [31]	Patients with ≥2 episodes in the previous 3 years	Penicillin V 250 mg BID (vs. placebo)	12 months	Recurrence: 22% vs. 37% (HR 0.55, 95% CI 0.35–0.86, *p* = 0.01)	45% risk reduction; no significant benefit in chronic edema, BMI ≥ 33 kg/m^2^, or ≥3 prior episodes
Kremer et al., 1991, J Infect *n* = 32 [26]	Patients with ≥2 episodes in the previous year	Erythromycin 250 mg BID (vs. no prophylaxis)	18 months	0/16 relapses vs. 8/16 (50%) in controls (*p* < 0.001)	Open-label RCT; 3 pts (20%) developed GI side effects → discontinued treatment
Olszewski et al., 2021, Lymphat Res Biol [28]	Patients with limb lymphedema and recurrent dermato-lymphangio-adenitis	Benzathinepenicillin 1.2–2.4 million units IM every 14–21 days	Several years	Recurrence risk reduced by ~95%; excellent tolerability; no resistance observed	Observational, single-center; long-term prophylaxis in lymphedema
Cochrane Review (Dalal et al., 2017, CD009758) [25]	5 RCTs (*n* ≈ 513) of recurrent cellulitis	Oral or IM penicillin, erythromycin	6–18 months	Pooled RR 0.46 (95% CI 0.26–0.79): prophylaxis effective during active treatment	Benefit lost after discontinuation; uncertainty about long-term efficacy, cost-effectiveness, and resistance

**Table 6:** Limitations and challenges of antibiotic prophylaxis in recurrent erysipelas.

Limitation	Details/Examples	Clinical Implications
Adherence issues (oral regimens)	Daily dosing with penicillin V, macrolides, or cephalosporins may be inconvenient; reduced compliance in patients with polypharmacy or malabsorption	Decreased effectiveness, risk of recurrence during/after treatment
Injection-related complications	Intramuscular benzathinepenicillin convenient (every 2–4 weeks), but carries risk of hematoma, especially in patients on anticoagulants (e.g., atrial fibrillation, obesity-related thromboembolism)	May limit use in elderly and high-risk patients
Gastrointestinal side effects	Nausea, diarrhea, abdominal pain, especially with macrolides or higher penicillin doses in obese patients	Reduced tolerance and adherence
CDI	Long-term antibiotic exposure increases risk of CDI	Potentially severe complication; requires careful risk–benefit assessment
Microbiota disruption & resistance	Selective pressure → colonization with MRSA, resistant Enterobacteriaceae	Reduced future treatment options, public health concern
Rising macrolide resistance	Resistance to erythromycin/azithromycin exceeds 20–30% in parts of Europe; higher rates in pediatric isolates	Limits usefulness of macrolides as alternatives to penicillin
Drug–drug interactions	Macrolides interact with statins, anticoagulants, QT-prolonging drugs	Requires regular medication review
Obesity-related reduced efficacy	Higher BMI often necessitates increased penicillin doses (e.g., ≥500 mg bid if BMI ≥ 33), yet prophylaxis may still fail	Obesity management is crucial adjunct to prophylaxis
Limited evidence for optimal duration	Studies suggest 6–18 months; relapse common after discontinuation; some guidelines recommend lifelong prophylaxis in high-risk patients	Individualized treatment duration and regular reassessment needed
Over reported penicillin allergy	Up to 90% of self-reported allergies not confirmed after testing; many patients can safely tolerateβ-lactams	Mislabeling leads to unnecessary use of macrolides, fueling resistance and reducing efficacy

**Table 7:** Adverse effects and limitations of antibiotic chemoprophylaxis in recurrent erysipelas.

Category	Issue	Examples/Details
Gastrointestinal intolerance	More frequent at higher doses, particularly in obese patients requiring double dosing	Nausea, diarrhea, abdominal pain; adherence problems [7]
CDI	Disruption of intestinal microbiota	Highest risk with clindamycin, moderate with macrolides/penicillins, low with tetracyclines [32]
Drug-specific toxicities	Cardiac, hepatic, hematologic, dermatologic adverse effects	Erythromycin: QT prolongation, arrhythmia, agranulocytosis [33]; Azithromycin: rash, cholestasis [34]; Clarithromycin: candidiasis, leukopenia, QT prolongation [35]; Doxycycline: photosensitivity, contraindicated in pregnancy [36]
Antimicrobial resistance	Rising macrolide resistance in *S. pyogenes*	<5% in some northern European countries vs. 20–40% in southern Europe [37,38,39,40]
Ecological impact	Selection pressure → multidrug-resistant organisms	Colonization with MRSA, resistant Enterobacteriaceae [7]
Penicillin allergy (often over reported)	Up to 90% of patients with reported allergy can tolerateβ-lactams	Allergy testing and carefulhistory recommended before switching to alternatives [23,24]
Drug–drug interactions	Increased toxicity when combined with anticoagulants or other drugs	Erythromycin ↔ apixaban/rivaroxaban (↑ bleeding risk) [33]
Route of administration	IM injections vs. oral dosing	Benzathinepenicillin IM: convenient monthly schedule but risk of hematoma in anticoagulated patients [41]; Oral: daily dosing, adherence issues, absorption problems

## Data Availability

All data supporting this study are from previously published sources, which are cited throughout the manuscript and available in public repositories such as PubMed. During the preparation of this manuscript, the authors used ChatGPT (GPT-5, OpenAI) for the purpose of creating figures.

## Abbreviations

The following abbreviations are used in this manuscript:

BID	lat. bis in die (eng. twice daily)
BLS	British Lymphology Society
BMI	Body Mass Index
BPG	Benzathine penicillin G
CDI	Clostridioides difficile infection
COPD	Chronic Obstructive Pulmonary Disease
CRP	C-reactive protein
ECG	Electrocardiogram
GAS	Group A streptococcus
GCS	Graduated compression stockings
HR	Hazard ratio
IDSA	Infectious Diseases Society of America
MRSA	Methicillin-Resistant *Staphylococcus aureus*
NICE	National Institute for Health and Care Excellence
NOAC	Novel Oral Anticoagulant
SSTI	Skin and soft tissue infection
TMP-SMX	Trimethoprim–sulfamethoxazole
RCT	Randomized Controlled Trial
